# Light-inducible genetic engineering and control of non-homologous end-joining in industrial eukaryotic microorganisms: LML 3.0 and OFN 1.0

**DOI:** 10.1038/srep20761

**Published:** 2016-02-09

**Authors:** Lei Zhang, Xihua Zhao, Guoxiu Zhang, Jiajia Zhang, Xuedong Wang, Suping Zhang, Wei Wang, Dongzhi Wei

**Affiliations:** 1 State key Lab of Bioreactor Engineering, New World Institute of Biotechnology, East China University of Science and Technology, Shanghai 200237, China; 2 College of Life Science, Jiangxi Normal University, Nanchang 330022, China; 3 Research Center for Biomass Energy, East China University of Science and Technology, Shanghai 200237, China

## Abstract

Filamentous fungi play important roles in the production of plant cell-wall degrading enzymes. In recent years, homologous recombinant technologies have contributed significantly to improved enzymes production and system design of genetically manipulated strains. When introducing multiple gene deletions, we need a robust and convenient way to control selectable marker genes, especially when only a limited number of markers are available in filamentous fungi. Integration after transformation is predominantly nonhomologous in most fungi other than yeast. Fungal strains deficient in the non-homologous end-joining (NHEJ) pathway have limitations associated with gene function analyses despite they are excellent recipient strains for gene targets. We describe strategies and methods to address these challenges above and leverage the power of resilient NHEJ deficiency strains. We have established a foolproof light-inducible platform for one-step unmarked genetic modification in industrial eukaryotic microorganisms designated as ‘LML 3.0’, and an on-off control protocol of NHEJ pathway called ‘OFN 1.0’, using a synthetic light-switchable transactivation to control Cre recombinase-based excision and inversion. The methods provide a one-step strategy to sequentially modify genes without introducing selectable markers and NHEJ-deficiency. The strategies can be used to manipulate many biological processes in a wide range of eukaryotic cells.

Filamentous fungi have a long-standing reputation as industrial producers of enzymes and metabolites, such as *Hypocrea jecorina* (Anamorph *Trichoderma reesei*). *H*. *jecorina* plays an important role in the production of plant cell-wall degrading enzymes^1^. Initially, scientists selected production strains from natural isolates that fulfilled both microbiological and technical criteria for economical production. Subsequently, genetically modified strains with novel properties and higher enzyme yields were obtained through traditional random mutagenesis^2^. In recent years, however, recombinant technologies have contributed significantly to improved enzymes production and system design of genetically manipulated strains.

Gene replacement via disruption, deletion and overexpression of target genes is a significant genetic tool for gene functional analysis. However, it is limited by the number of available selectable marker genes. In recent years, a Cre/*loxP* recombination system has been widely used for marker rescue in various organisms. With the Cre/*lox*P system, genetic modifications were successfully introduced into different organisms by recycling a single selectable marker. Cre-mediated recombination depends on the expression of *cre*. Retransformation with a *cre* gene that remained in the fungal genome^3^,^4^ is associated with the risk of chromosomal rearrangement and Cre toxicity^5^. Another method is transient expression of Cre recombinase by Florea *et al.*^6^. The disadvantage of this method is low efficiency (marker was eliminated in 0.5–2% of the colonies) and two-step processes including fungal transformation and transient transfection. A third option is direct introduction of Cre protein, which is still a time-consuming two-step process^7^. A fourth option is PCR-based fusion of self-excision cassette, which includes a series of line DNA fragments fused by PCR, and PCR-generated errors might be introduced into the genome^8^. Another potential disadvantage of this method is that the gene disruption cassettes they generated are linear DNA fragments by PCR. They are not circular plasmids which can easily be transformed into bacterial cells and purified away from them.

Furthermore, previous methods above cannot easily transplant from one fungus to another one, because different systems using different inducible promoters. Native fungal inducible systems with an inducible promoter from one fungus strain are not always well recognized or regulated by another system. Therefore, engineered regulatory systems are good choices for *cre* expression in a wide range of eukaryotic cells. Engineered regulatory systems to control fungal protein expression include chemically regulated gene expression systems that respond to orthogonal molecules such as estrogen (Gal-ER-VP16)^9^ or doxycycline (Tet-OFF)^10^, and light-inducible transcription systems^11^,^12^,^13^. Chemical inducers of protein dimerization have been extensively used to allow inducible control of signal transduction pathways and transcription in live cells. However, the systems are affected by compound uptake or drug pump issues, which can confound studies. For example, using Gal-ER-VP16 system protein levels are controlled by the addition of β-estradiol to the media^9^ resulting in considerable differences in the rate of β-estradiol uptake, with different strains or gene deletions that affect membrane transporters or drug efflux pumps drastically altering reporter protein expression levels. For instance, the Gal-ER-VP16 system does not work in a strain that cannot use β-estradiol. In contrast to chemicals, light is an ideal inducer of gene expression because it is highly tunable and has high spatiotemporal resolution^11^,^12^,^13^.

Homologous recombination (HR) and non-homologous DNA end-joining (NHEJ) both play critical roles in repairs of double-strand DNA breaks (DSBs). In the unicellular eukaryote *Saccharomyces cerevisiae*, HR is the main role involved in repair of DSBs, whereas NHEJ is the most active damage response mechanism in multicellular eukaryotes^14^. HR is, in principle, the most efficient method of disrupting, modifying, or replacing a target gene. Unfortunately, in most multicellular eukaryotes, the gene targeting efficiency is quite low because of the predominance of NHEJ over HR pathway^15^. We considered that these two repair pathways work independently and competitively^16^. Consequently, a strategy to eliminate or inhibit the NHEJ pathway is needed to achieve highly efficient gene targeting in multicellular eukaryotes.

In eukaryotes, the NHEJ system is composed of the DNA-dependent protein kinase catalytic subunit (DNA-PKcs), the DNA ligase IV-Xrcc4 complex and the KU70-KU80 heterodimer (KU complex)^15^,^17^. The NHEJ occurs in bacteria, fungi and mammals, indicating evolutionary conservation^17^. In *Neurospora crassa*, the HR efficiency increased to 100% in the KU disruption strains, compared to 5 to 20% in the wild-type species^18^. This approach has proven to be a major gene targeting breakthrough in most filamentous fungi. Deletion of the KU70 or KU80 gene to eliminate the NHEJ activity has dramatically increased the frequency of HR in numerous filamentous fungi such as *Aspergillus nidulans*, *A. sojae*, *A. oryzae*, *A. niger* and *H. jecorina* (Anamorph *Trichoderma reesei*)^19^,^20^,^21^,^22^.

However, deficient KU70/KU80 orthologues in different fungal species triggers varying susceptibility to different DNA damaging agents. For example, *A. fumigatus* showed mild sensitivity towards methyl methanesulfonate (MMS)^23^. In contrast, *N. crassa* showed increased sensitivity towards phleomycin and MMS^24^. A higher UV sensitivity was reported for *A. niger*^21^ and *H. jecorina* Δ*tku70* strains^22^. Such strains deleted in KU70/KU80 orthologues lack an important DNA repair mechanism. Furthermore, KU complex affected fundamental cellular aspects such as telomere maintenance, nuclear spatial organization or mitotic recombination^25^,^26^. To minimize the effect of a *ku70*/*ku80* deletion, it is very advantageous to restore the NHEJ pathway by reintroducing the *ku*70/*ku80* gene. The Δ*ku70* strain can be retransformed with endogenous *ku70*. But, another genetic modification is necessary to screen for integration of the *ku70* gene retransformation. Another possible method involves conventional genetics by crossing out the *ku70*/*ku80* deletion, especially in strains already subjected to sexual crossing. However, sexual crossing is time-consuming, and most industrially relevant fungi do not have a sexual cycle. A third option presented by Nielsen *et al.*^27^ used a transient disruption of *nkuA*, KU70 orthologue in *A. nidulans*, which was rectified by removing the bifunctional marker gene (*pyrG*) flanked by a direct repeat and re-establishing a functional *nkuA* gene spontaneously. A last option presented by Janus *et al.*^28^ involves transient silencing of the *Pcku70* gene based on an autonomously replicating AMA1-based RNAi plasmid. However, the targeting efficiency obtained was less than in a Δ*Pcku70* strain^28^. In addition, AMA1-bearing self-replicative plasmids undergo rearrangement and multimerization at higher frequencies in some species^29^.

For efficient and sequential gene modification, we leveraged the widely used Cre/*loxP* recombination system, which can switch on gene expression by DNA excision and inversion^30^. We designed a one-step method to combine self-excisable marker rescue and an on-off control NHEJ pathway in industrial eukaryotic microorganisms.

In our system, *cre* was transiently expressed to generate marker-free transgenic fungi. Disrupted KU70 function was rapidly switched repeatedly using a simple selection scheme for the desired genetic manipulations. This controllable genetic manipulation is a powerful and labor-saving tool in fungal strains and easily fulfills the essential safety requirements to obtain marketing approval.

## Results

### Design of LML 2.0

**Figure 1:**
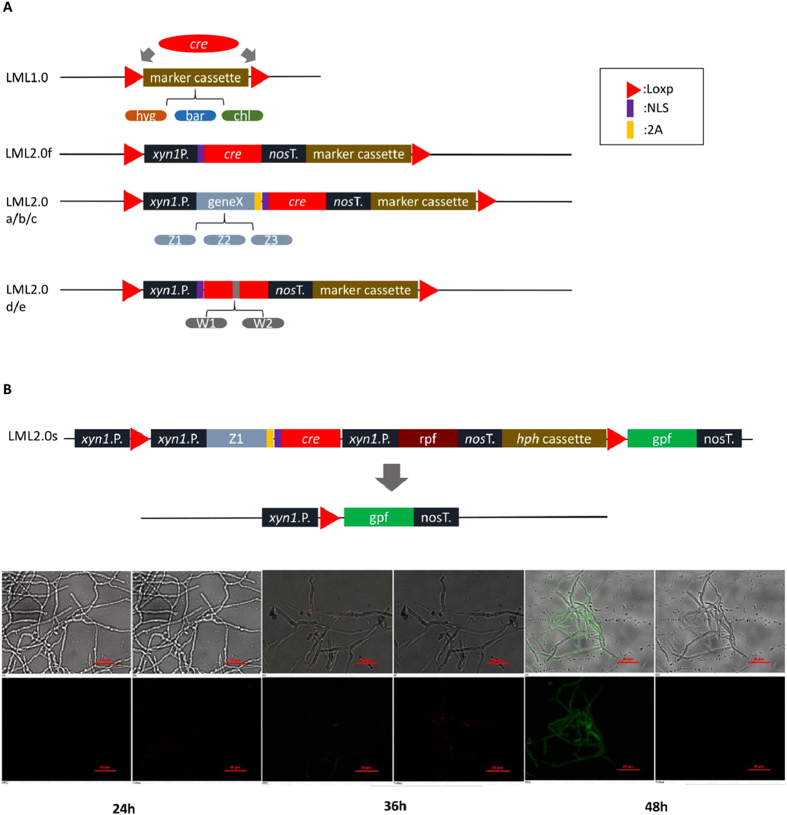
Construction of LML cassettes. (**A**) LML 1.0 and 2.0 cassettes. (**B**) Multicolor labeling cassette LML 2.0s and fluorescence micrographs of marker self-excision course using fluorescence microscopy in *H. jecorina* transformed with LML 2.0s. Bars represent 20 μm.

Currently, several methods have been reported for unmarked genetic modification of eukaryotic microorganisms. However, these methods were tedious and entailed a two-step process^3^,^4^,^6^,^7^,^8^. Our objective was to upgrade from the previous LML1.0^3^,^4^ to a new self-excisable gene disruption system, LML 2.0, which spliced two *loxP* sites, a Cre expression cassette and a selectable marker together to generate multiple fungal mutants through efficient one-step recycling of markers. This method obviates the need for a heterologous Cre expression cassette or Cre protein. It is much faster and more efficient than current gene deletion methods in fungi, and reduces Cre toxicity. It could also be used sequentially to delete fungal genes without introducing selectable markers. Unfortunately, no stable plasmid was available for LML 2.0f design (Fig. 1A) in an *E. coli* host. We observed sufficient Cre activity from the *xyn1*-*cre* construct to catalyze the recombination of *loxP* plasmids in *E. coli,* confirming that many eukaryotic promoters were active in bacterial cells.

To prevent the bacterial expression of *cre* driven by fungal promoters, we initially constructed three chimeric *cre* genes (*z1cre*, *z2cre* and *z3cre*). Three randomly selected predicted genes (*z1*, *z2* and *z3*) from the *H. jecorina* were fused with the *cre* coding sequence by a 2A peptide, respectively. This 2A peptide could generate two mature proteins by self-cleavage. One protein is *z1*, z2 or *z3* encoding peptide. The other one is Cre recombinase. Second, we constructed two modified *cre* genes (*w1cre*, *w2cre*), in which the coding sequence of native *cre* was interrupted by the two introns from the *H*. *jecorina cbh1* gene, respectively. The resulting fungal transformation vectors LML 2.0a–e are shown in Fig. 1A. Restriction analysis and PCR demonstrated the absence of any excision from these plasmids in both *E. coli* and *A. tumefaciens* demonstrating that intron insertion abrogated all enzymatic activity of the *cre* gene in bacterial cells.

Using the *Agrobacterium*-mediated transformation, the above constructs were introduced into *H*. *jecorina* Qm6a, *N. crassa* and *A. niger*. Eight hygromycin B-resistant transformants for each host (*H. jecorina* Qm6a, *N. crassa* and *A. niger*) were randomly selected. These transformants were confirmed to have an intact LML 2.0 by PCR and a single copy of LML 2.0 by qPCR. The modified *cre* genes were drived by the *xyn1* promoter which was induced by xylose. Introns were recognized and excised during the transcription to produce Cre recombinase that affected two *loxP* sites in the same orientation. For *z1cre*, *z2cre* and *z3cre*, the 2A peptide underwent self-cleavage to generate the mature *H. jecorina* protein Z1 (Z2, or Z3) and Cre recombinase, respectively.

As shown in Table 1, LML 2.0a–e auto-excised in these 3 strains, and the average self-excision efficiencies were 60 to 85% for a, b and c, 20 to 90% for d and e. This *xyn1*-regulated LML 2.0 system works in *A. niger* as well and is also successfully transferred into *N. crassa*, leading to self-excision of the *loxP* maker cassette. These findings point to the general applicability of this system in filamentous fungi. Furthermore, the self-excision efficiencies of LML 2.0a–c seem to be slightly better than 2.0d and e due to stable self-excision efficiency in the 3 strains. The introns, obtained from *H. jecorina cbh*1, inserted into *w1cre* and *w2cre*, were not native, while the introns in *z1cre*, *z2cre* and *z3cre* were originally derived from *z1*, *z2* and *z3*.

The *xyn1* promoter is active in the presence of xylan and D-xylose, and virtually silenced in the presence of glucose. Therefore, the *xyn1* promoter controlled LML 2.0 system well. However, previous reports^31^ showed that industrial *cre*1-deficient strain RUT C-30 exhibited a basal level of *xynl* expression on glucose much higher than that of *cre*1 intact strain QM6a and QM 9414. To investigate if LML 2.0 was stable enough for industrial *cre*1-deficient strains, we analyzed the self-excision average efficiencies of 2.0a with different carbon source in *cre*1 deficient strain RUT C-30. The results (Table 2) showed that LML 2.0a had much higher self-excision efficiencies with D-xylose than other D-xylose downstream metabolites, xylitol, L-arabinose and L-arabitol. These metabolites failed to induce *cre* expression under the control of *xyn1* promoter as the self-excision efficiencies were lower than that of glycerol, a repressing carbon source. Our data showed that the induction efficiency of L-arabitol on *cre* expression under the control of *xyn1* promoter was lower than that of glycerol. This result is, however, in conflict with published reports^32^,^33^, which suggested that L-arabitol as well as D-xylose also induce *xyn1* promoter transcription in *H. jecorina*. Transcript analysis was based on a single time point under induction by D-xylose downstream metabolites and was therefore over-estimated. The *xyn1* promoter used to control *cre* expression was truncated (0.6 kb) and not in *xyn1* locus.

The results (Table 2) also showed that LML 2.0a was quite stable under repressed conditions (with glucose as carbon source) in wild-type strain Qm6a and even industrial *cre*1-deficient strain RUT C-30. However, LML 1.0 is not so stable under glucose conditions in RUT C-30 (self-excision strains (total strains): 14(96)). To investigate the factors underlying the different stabilities under repression, a red fluorescent protein was fused behind *z1cre* and *cre*, which were integrated into *xyn1* locus instead of *xyn1* gene, respectively (Fig. S-1). The fluorescence intensity data (Fig. S-1) showed that the expression of *z1cre-rfp* in cell nucleus was much lower than that of *cre-rfp* although they were under the control of the same *xyn1* promoter. A reasonable explanation for this is that *z1* gene fused before *cre* had a negative effect on the expression levels of functional Cre recombinase. A lower Cre recombinase leads to lower Cre toxicity, lower efficiency of self-excision, and higher stability under repressed condition.

In order to visualize the process of self-excision, multicolor labeling cassette LML 2.0s was constructed and shown in Fig. 1B. The *H. jecorina* transformants were used to analyze fluorescence during the growth after 24, 36, and 48 hours on Mandels’ medium (MA) containing D-xylose. The time course of changes in color from red to green, represented the course of self-excision (Fig. 1B). The excision above was tested with PCR and gene sequencing for the related gene locus. Finally, we used phosphinothricin acetyltransferase and sulfonylurea resistance allele expression cassette instead of hygromycin B phosphotransferase to construct plasmids, LML 2.0a-bar and 2.0a-sur. We confirmed that this *xyn1*-regulated LML 2.0a system works in *M. anisopliae*, which led to the ~83% self-excision of the LML cassette.

### Design of LML 2.1 cassette

**Figure 2:**
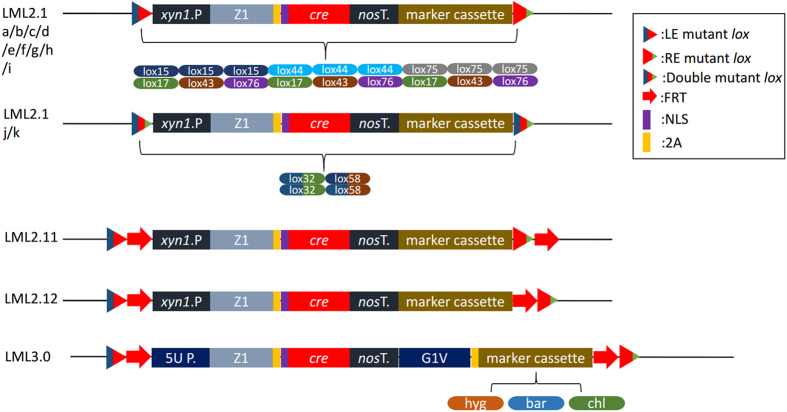
Construction of LML 2.1, 2.11, 2.12 and 3.0 cassettes.

One more *loxP* site was added to the genome during each round of genetic modification and subsequent marker self-excision using the LML (1.0 or 2.0) system, which was associated with the risk of chromosomal rearrangement between the remaining *loxP* sites when carrying out the marker recycling process^34^. Therefore, we upgraded from LML2.0a to LML2.1, which employed the LE/RE mutant strategy using LE mutant *lox* carrying mutations in the left-inverted repeat region and RE mutant *lox* carrying mutations in the right-inverted repeat (Fig. 2). Recombination between a LE mutant *lox* and a RE mutant *lox* resulted in the generation of a double mutant *lox* site with mutations at both ends (Fig. 2). The Cre recombinase cannot efficiently catalyze recombination between the double mutant *lox* sites. The recombination reaction between double mutant *lox* sites was unavailable based on transient expression of Cre proteins by our LML 2.1 for only a limited time before its self-excision. Therefore, chromosomal rearrangement rarely occurred between the double mutant *lox* sites in the genome during further rounds of marker rescue.

We selected three LE *lox* sites (*loxJT15*, *lox44* and *lox75*) and three RE *lox* sites (*loxJTZ17*, *lox43* and *lox76*)^35^. To test whether these mutant *lox* sites hindered recombination efficiency or promoted stability in our system, we used the same strategy as LML 2.0a to build LML 2.1a–k (Fig. 2).We constructed nine integration cassettes, LML 2.1a–i, harboring different LE/RE mutant *lox* pairs. *H. jecorina* transformation was followed by comparison of self-excision frequencies (Table 3). The average frequency of the LML 2.1a, *loxJT15*/*loxJTZ17* pair, was 92%, which was higher than that of 2.0a (~80%). The frequency of LML 2.1b, *loxJT15*/*lox43* pair was 26%, and the remaining pairs showed similar and lower frequencies of about 2–10%. We then synthesized two double mutant *lox* sites, *lox32* (recombination between *loxJT15* and *loxJTZ17*) and *lox58* (recombination between *loxJT15* and *lox43*) and established LML 2.1j and k with them, respectively. The average frequency of the LML 2.1j was 2% and that of 2.1k was 0%, which were much lower than that of wild-type *loxP* (60%). The results show that LML 2.1a and b were suitable for sequential gene modification, and too stable to induce recombination when carrying out further marker recycling processes.

The *tku70* deletion cassette was used to transform *H*. *jecorina* Qm6a. The Qm6aΔ*tku70* strain with correct transformation and marker excision was confirmed using PCR and qPCR. We used Qm6aΔ*tku70* as a host strain to delete several genes. We performed six additional deletions and determined homologous integration and self-excision frequencies (Table 4). PCR and gene sequencing of the gene-targeting locus in the excision strains revealed one remaining double mutant *lox32* at the locus.

As expected, the Qm6aΔ*tku70* strain showed similar homologous integration rates (Table 4). However, the hygromycin B excision frequencies were quite different between genes (Table 4). No self-excision was detected in Δ*xyr1* strain for the general activator Xyr1 as essential for *xyn*1 transcription. The expression of *z1cre* was under the control of *xyn1* promoter, which was governed by transcription factors *xyr1* and *ace2*. However, few self-excision strains were detected in Δ*xyr*1 strain using 2.11 and 2.12 cassettes (Table 4). Very few self-excision strains of LML 2.0a or 2.1a in Δ*xyr*1 strain were detected after two or three rounds of excision (Table 4). Consistent with earlier reports^36^, our results clearly demonstrate that the *lox*-*FRT* fusion sequences as recognition sites dramatically enhance the Cre-mediated excision efficiency. Luo *et al.*^36^ speculate that the fused *loxP*-*FRT* sequences may enhance the alignment of the recognition sequences, DNA bending or cleavage, or the formation of a Holliday junction or DNA–recombinase complex, resulting in improved efficiency. A 86-bp-length *lox*-*FRT* site scar was left after each round of self-excision of their ’GM-gene-deletor’ system^36^. Similar results were obtained with our LML 2.11. The LML 2.12 cassette, including one *loxJT15*-*FRT* site and one *FRT*-*loxJTZ17* site, has never been reported before, with excision efficiency similar to that of LML 2.11. Furthermore, self-excision resulted in a scar of 34-bp-length *lox32*, shorter than 86-bp-length *lox32*-*FRT* of LML 2.11. The results encourage us to upgrade our one-step marker introduction/self-excision system for fungal strains, with disrupted xylanase regulators, or in which *H*. *jecorina xyn1* promoter was not recognized or regulated.

### Design of LML 3.0 cassette

Compared to LML 2.1, 2.11 and 2.12, we anticipate that the LML 3.0 is able to efficiently excise in other fungi subjected to sequential gene modification. Native fungal inducible systems from one fungus strain are not always well recognized or regulated by another system, just as *xyn1* promoter was incompatible in Δ*xyr1* strain above. Therefore, we upgraded the LML 2.12 to 3.0 using a light regulator to control *cre* expression. Light-inducible transcription systems compatible with LML 3.0’s that work with different types of fungal hosts (other than 2.0 and 2.1) need to be designed.

Recently, we described a blue light-mediated regulation of DNA transcription in filamentous fungus *T. reesei*^37^. Using light-switchable transactivator G1V and its binding promoter 5Up^37^, we constructed LML 3.0 (Fig. 2) and LML 3.0s (Fig. S-2). The two active sites included *loxJT15*-*FRT* and *FRT*-*loxJTZ17* fusion sequences. Using *Agrobacterium*-mediated transformation, the LML 3.0 constructs were introduced into *H. jecorina* Qm6a, Qm6aΔ*xyr*1, *N. crassa*, *A. niger and M. anisopliae*. Using hygromycin B, phosphinothricin or chlorimuron ethyl, transformants for each cassette were randomly selected. These strains express Cre recombinase under the control of light. After the light-inducible self-excision, the phenotype was tested on plates containing corresponding antibiotics. As shown in Table 5, approximately 40–70% of the transformed strains had successfully excised the LML 3.0 cassette and did not show growth on corresponding antibiotic-containing plates. LML 3.0 excised in all the five strains, including Δ*xyr*1 strain and even yeast strains (unpublished work). The LML 3.0 cassettes were widely used in more hosts than LML 2.0/2.1 due to artificial light-inducible control of self-excision.

### Design of OFN 1.0

NHEJ-deficient strains may negatively influence genome stability and fitness of the respective strains. For example, this mutation causes telomere shortening^38^, increased sensitivity to DNA damage^26^, higher susceptibility to various toxins and irradiation^21^. To avoid such drawbacks and to minimize the effect of a *ku70* deletion, four main strategies are used currently to restore the NHEJ pathway: (1) ectopic or homologous integration of the endogenous *ku70* gene; (2) sexual back-crossing; (3) transient disruption based on direct-repeat recombination^27^; and (4) transient expression system with an inducible RNAi vector^28^. The disadvantages include: (1) time-consuming genetic modification to screen for integration of the *ku70* gene; (2) sexual back-crossing leads to additional undesired phenotype effects and is time-consuming. In addition, sexual crossing experiments cannot be performed with some asexual strains; (3) transient systems from Nielsen *et al.*^27^ have a low screening frequency and the strain restored *pyrG* negative, requiring uridine for cultivatation; (4) transient silencing system from Janus *et al.*^28^ have a targeting efficiency lower than that of a Δ*ku70* strain. The AMA1-bearing plasmids undergo rearrangement and multimerization at higher frequencies in some species^29^.

Clearly, a new strategy is needed for the rapid and accurate on/off control of NHEJ pathway. Here we describe a foolproof strategy to on/off control the expression levels of *tku70* in *H. jecorina* using the Cre/*loxP* system. DNA sequence flanked by *loxP* sites can be inverted or excised in the presence of the Cre recombinase, which depends on the orientation of the two *loxP* sites. We deduced that the orientation of *tku70* gene would continuously be converted by Cre recombinase catalyzing, because *tku70* gene was flanked by two inverted *loxP* sites. If Cre recombinase was removed from the cell, *tku70* gene in one cell nuclei would be in one stable orientation. By adding the Cre recombinase self-excision system, Cre recombinase was removed from the cell. Finally, we can obtain two kinds of cell nuclei with two orientation of *tku70* gene. We can get two strains including two orientation of *tku70* gene by single spore isolation, respectively.

**Figure 3:**
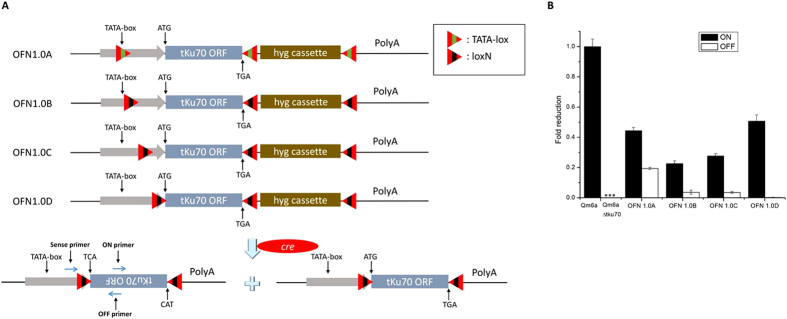
An on-off control protocol of nonhomologous end-joining (NHEJ) pathway using the Cre-lox system. (**A**) Construction of OFN 1.0A–D cassettes. Genomic PCR of multiple strains using a given primer configuration (left bottom) shows inversion only after Cre induction. (**B**) Comparative transcript ratio analysis of *tku70*. Transcript ratios for ON and OFF state were calculated using ABI Stepone plus software. Values above 1 indicate higher transcription in the ON state strain compared to QM6a, and values below 1 indicate lower transcription. Error bars represent 95% confidence intervals. ***means not done.

We developed and tested cassettes (OFN 1.0 system, Fig. 3) that incorporated two 2-state designs where *tku70* adopted either A–C (LOW and HIGH) or D (OFF and ON) constitutive expression states after “inversion” with transient Cre expression of LML 2.0 or more advanced system. The *loxP* sites of OFN 1.0 and LML system may lead to irreversible recombination exacerbated by additional genetic modification. To address this issue, we utilized orthogonal *loxP* sites (commonly referred to as *loxN* and TATA*lox*) (Fig. 3A) that are capable of recombining with themselves, but do not interact with *loxP* sequences or each other.

**Figure 4:**
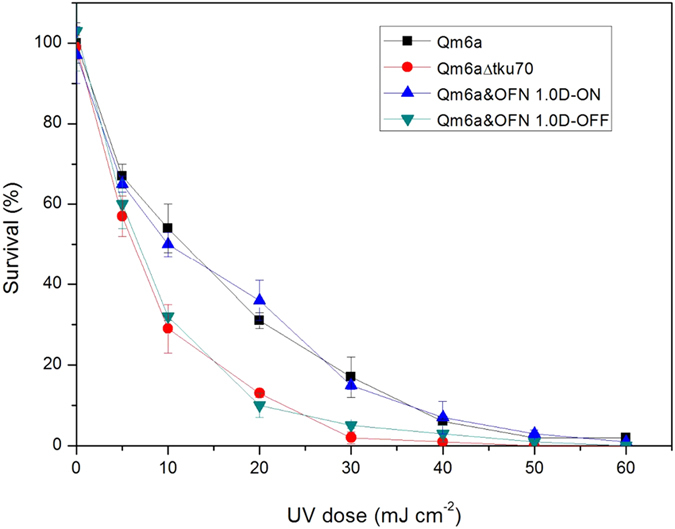
Survival rate of of Qm6a, Qm6a*Δtku70*, Qm6a&OFN1.0D-ON and Qm6a&OFN1.0D-OFF strains following exposure to UV. Spores were exposed to different doses of UV. Aliquots of the UV-irradiated spores were plated on potato dextrose agar plates containing the colony restrictor Triton X-100 and the surviving colonies were counted.

We constructed 4 cassettes (OFN 1.0A–D) in which the gene regions of *tku70* were flanked with inverted *lox* sites (Fig. 3A). In OFN, 1.0A–D *tku70* was flanked by inverted TATA*lox* or *loxN* sites at different loci (from TATA box to -1 of *tku70* promoter), which alter its expression by inversion (Fig. 3A). The Cre recombinase transiently expressed from LML system can invert two *lox* sites of OFN 1.0 and transform ON state into OFF state. To test this randomization strategy, we used a PCR strategy to verify promoter inversion (Fig. 3A), and qRT-PCR to analyze *tku70* transcription (Fig. 3B). We observed a 50%/50% split in *tku70* ON and OFF state after excision of the *lox*P marker cassette, and obvious *tku70* transcription in ON state strains (Fig. 3B). We detected lower transcript levels of *tku70* in OFN 1.0A–C OFF states (Fig. 3B); however, it was barely detectable in 1.0D OFF state (Fig. 3B). Increased tolerance of UV irradiation was seen in ON state strains compared to the OFF state strains and Δ*tku70* strains (Fig. 4). The results showed that *tku70* were restored by inversion, though the *tku70* transcriptional level in OFN 1.0D ON state is lower than that in wild type strain.

## Discussion

The development of a genetic system that allows highly efficient homologous integration with resilient NHEJ deficiency and simultaneous transgene removal using light, is of great significance in the regulation of transgene expression in filamentous fungi.

In this paper, we have presented a new Cre/*loxP*-based system to generate marker-free transgenic fungi, with high self-excision efficiency, via a single-step transformation. Cre recombinase mediated simultaneously the elimination of the selectable marker gene and *cre* gene. Antibiotic susceptibility, PCR screening and integration border sequencing demonstrated unequivocally that the excision led to marker-free fungi strains. Unlike the fungal Cre/*loxP* system described previously, this system has several characteristics as follows.

First, the structure of our LML 2.0 or upper system is integral and simplified. Our system obviates the need for introduction and elimination of a heterologous Cre expression plasmid and is much faster than current gene deletion methods in filamentous fungi. We created three chimeric *cre* genes (*z1cre*, *z2cre* and *z3cre*) to prevent the bacterial expression of *cre* driven by fungal promoters and constructed this all-in-one cassette (*loxP*-*cre*-Marker-*loxP* region). The three genes (*z1*, *z2* and *z3*) were randomly selected from *H. jecorina*. Selection of a highly conserved gene element, such as chromodomain^39^ encoding gene, enables construction of chimeric *cre* gene, for transformation of any eukaryotic cell. The system utilizes the conventional *Agrobacterium*-mediated transformation. All unnecessary components including *cre* gene and selectable marker gene are moved from transgenic fungal genome after induction. The transient expression of the *z1cre* gene prior to self-excision as established here prevents accumulation of large amounts of toxic Cre protein.

Second, our system employs the LE/RE mutant *loxP*. Recombination of *loxJT15* and *loxJTZ17* sites results in a double-mutant *lox32* site (Fig. 1). The *lox32* site left in chromosomes inhibited excision events and thereby maintained genomic stability, since it is not effectively recognized by Cre recombinase.

**Figure 5:**
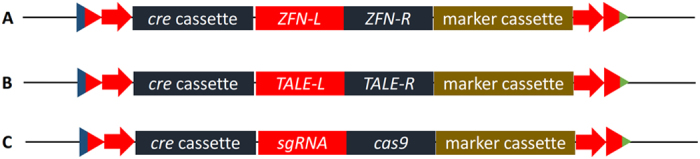
Relationship between LML 2.0/2.1/3.0 system and ZFNs (**A**), TALENs (**B**) or CRISPR (**C**) system.

Third, our system uses circular plasmids, which can be preserved in bacterial cells and are easy-to-use, as *cre* gene is interrupted by introns to prevent the bacterial expression of *cre* driven by fungal promoters. Finally, expression of *cre* gene is induced by blue light, which is of utmost importance since *cre* gene placed under the control of an inducible promoter *xyn1* from *H. jecorina* cannot be widely recognized by other strains, such as *xyr1* deficient host. The LML 3.0 system has been shown to be functional in all five filamentous fungi in this study but is likely to be applicable to other eukaryotic cells as well, such as yeasts and plants. The LML 2.0/3.0 system potentially acts with zinc finger nucleases (ZFNs), transcription activator-like effector nucleases (TALENs) or short palindromic repeat (CRISPR) system (Fig. 5).

Furthermore, we describe an on-off control protocol of NHEJ pathway called ‘OFN 1.0’ in *H. jecorina*. This is a key experimental model for genetic and metabolic regulation of enzyme production^40^,^41^,^42^,^43^. To address the limitations of using NHEJ-deficient strains, we have demonstrated rapid and repeated randomization of the two expression states (ON or OFF) of *tku70* in *H. jecorina* by flanking the gene with inverted *loxP* repeats. Using orthogonal *loxP* sites (*loxN* and TATA*lox*), the OFN and LML systems coexist in the same genetic background. Using a single-step of blue light illumination, the self-excision of marker and *cre* genes and the restoration of NHEJ pathway happen simultaneously. We suggest that the design of light-regulated OFN 1.0 system is also useful in other branched pathway. Using on-off or high-low control expression of proteins regulating the flux through alternate branches, the innate capabilities of the network to produce different outcomes or products can be explored.

In summary, we presented a foolproof integrated strategy to generate genetically engineered multicellular eukaryotes in gene targeting without NHEJ deficiency and to recycle the markers during the each transformation. It is possible to perform sequential steps of genetic modification without a shortage of markers. This strategy allows functional analysis of genes in a defined genetic background, including marker-free deletion and NHEJ pathway restoration. Furthermore, this strategy enables the creation of recombinant strains without possible disturbance of marker gene residual or NHEJ deficiency on the physiology or metabolism of the strains^26^,^42^. Our results demonstrate that an acceptable transgenic manipulation has been established to produce marker-free homologous recombination in eukaryotic cells, which can be used to produce safe transgenic organisms.

## Materials and Methods

### Strains and media

*Escherichia coli* DH5α was used as a host strain for the recombinant DNA manipulations. *H. jecorina* strains including wild type strain QM6a, mutant RUT C-30, *N. crassa* wild type strain FGSC2489 and *Metarhizium anisopliae* ARSEF2575, belonging to the *Sordariomycetes*, and *A. niger* wild-type strains^44^ were maintained on malt extract or potato dextrose agar. *Agrobacterium tumefaciens* GV3101 and AGL-1 were used in the *Agrobacterium*-mediated transformation system. Luria–Bertani (LB) medium was used for *E. coli* and *A. tumefaciens* culture. Mandels’ medium (MA)^45^ and M-100 medium^46^ were used for general fungal transformation. The fungal strains constructed in this study are summarized in Supplementary Table S-1.

### Plasmid construction

All the plasmids used in this study are listed in Supplementary Table S-2. The vectors were built using compatible cohesive ends generated by *Xba*I and *Spe*I in pPK2-derived BioBrick base vector, as previously described^47^. In this study, three kinds of marker cassettes, including hygromycin B phosphotransferase, phosphinothricin acetyltransferase and sulfonylurea resistance allele expression cassette were used to construct plasmids. The construction of LML 1.0, 2.0 (a–f, and s), 2.1 (a–k), 2.11, 2.12 and 3.0 is shown in Fig. 1A,B and 2.

The LML 1.0 cassette includes two repeated *lox*P sites with a marker in between them. The *cre* recombinase gene was inserted into the genome instead of *xyn*1 gene. In this study, *cre* was fused with a nuclear localization sequence (NLS; GenBank: EHK22773.1).

Compared to LML 1.0, LML 2.0a–c have an additional Cre recombinase expression sequence, which includes a *xyn*1 promoter, a native gene (*z1*, *tre111731*; *z2*, *tre112680*; or *z3*, *tre112518*) from *H. jecorina*, a 2A self-cleavage peptide^48^, *cre* gene fused with NLS and a *nos* terminator (GenBank: KF499077.1), between the two repeated *loxP* sites. In LML 2.0d and 2.0e, the Cre recombinase includes a modified *cre* gene (*w1cre* or *w2cre*), in which *cre* coding sequence is interrupted by one of two introns from the *H*. *jecorina cbh1* gene (*tre123989*), respectively (unpublished work).

LML 2.0s cassette was constructed from LML 2.0a by adding a red fluorescent protein expression sequence, and a *xyn*1 promoter and a green fluorescent protein gene with a flanking *nos* terminator externally, respectively.

To construct LML 2.1a–k cassettes two *loxP* sites were replaced by different left element/right element (LE/RE) mutant *loxP* sites^49^. To construct LML 2.11 or 2.12 cassettes the left *loxP* sites were both replaced by *loxJT15*-*FRT*^50^ fusion site, and the right *loxP* sites were replaced by *loxJTZ17*-*FRT* or *FRT*-*loxJTZ17* fusion sites, respectively.

Compared to LML 2.12, LML 3.0 cassettes have a light-switchable transactivator G1V gene, which is fused with the selectable marker genes by a 2A peptide, and a corresponding light-switchable promoter 5Up for Cre recombinase expression instead of *xyn*1 promoter.

The *H*. *jecorina tku70* deletion vector was used in the pPK2-derived BioBrick plasmid and comprised the LML 2.1a cassette flanked by 2.0-kb fragments up- and downstream of *tku70*. The flanking region fragments were amplified by PCR using primer pairs KU705-F/R y KU703-F/R. The construction of cassettes for the 5 deletions (*cbh1*, *cbh2*, *tre108087*, *cre1*, *ace1*, *ace2*) in the *tku70* deficient strain was performed in the same BioBrick plasmid mentioned above and contained the LML 2.1a cassette flanked by 1-kb fragments up- and downstream of the target gene.

Four types of *xyr1* deletions in *H*. *jecorina tku70* deficient strain contained the LML 2.0a, 2.1a, 2.11 or 2.12 cassette, respectively, flanked by 1-kb fragments up- and downstream of *xyr1*. The flanking region fragments were amplified by PCR. Primers used for the 7 deletion constructs are listed in Table S-3 in the supplemental material.

In *H. jecorina* RUT C-30 strain, two chimeric genes, a red fluorescent protein gene fused behind *z1cre* and *cre*, respectively, were integrated into *xyn1* locus instead of *xyn1* gene (see Supplemental Material Fig. S-1).

The on-off control cassettes of NHEJ pathway, OFN 1.0A–D, are shown in Fig. 3A. To generate OFN 1.0A cassette three bifunctional TATA-*lox*^51^ sites and a hygromycin B marker self-excision cassette were inserted around *tku70*. The first reverse TATA-*lox* site was inserted 65 nt upstream of ATG of *tku70* by PCR-mediated mutagenesis instead of − 65 to −32 of *tku70* promoter. Therefore, the TATA box (− 52 to –38) of *tku70* promoter was replaced by the first bifunctional TATA-*lox* site. The second TATA-*lox* site was set behind TGA codon of *tku70*. The third TATA-*lox* site was behind self-excision cassette.

To generate OFN 1.0B–D cassettes three *loxN*^52^ sites were inserted around *tku70*. The first reverse *loxN* sites were all inserted downstream of TATA box sequences of *tku70* promoter in the three cassettes. However, they were located in different loci: −38 of *tku70* promoter for 1.0B; −30 for 1.0C; and −1 for 1.0D. The second and third *loxN* sites and self-excision cassette were located similar to TATA*lox* in 1.0A cassette.

### Transformation of T. reesei

Transformation of the hygromycin B resistance marker vectors was performed based on protocols described by Covert *et al.*^53^. Transformation of the phosphinothricin resistance marker vectors was performed based on protocols described by Zhang *et al.* (manuscript in preparation). Transformation of the chlorimuron ethyl resistance marker vectors was based on protocols described by Lin *et al.*^54^.

### Characterization of the transformants

For each vector transformation, 4 to 8 transformants of Δ*tku70* strains or 12 transformants were collected. We used qPCR to verify that a single copy from the expression cassettes was successfully integrated into the genome. Analysis of the integration of the target gene-deleted construct was performed using diagnostic PCR and qPCR.

### Excision of the loxP marker cassette

To excise the LML cassettes, transformants of LML 1.0, 2.0, 2.1, 2.11 and 2.12 were cultivated on an MA medium containing 1% (wt/vol) glycerol, glucose, D-xylose, xylitol, L-arabinose, or L-arabitol (Sigma Aldrich, St. Louis, MO) by shaking (200 rpm) at 28 °C for 2 days. LML 3.0 transformants were cultivated on an MA medium containing 1% (wt/vol) glucose exposed to 53 W/m^2^ constant blue light (460 nm peak) for 2 days. Conidia were harvested after 4 to 8 days under conditions similar to liquid culture before, plated on an MA agar plate containing 0.1% (wt/vol) Triton X-100 (Sigma Aldrich) and 1% (wt/vol) carbon sources. The phenotypes of the 8 to 24 fungal isolates of each transformant were monitored by plating them on an M-100 plate containing corresponding selection markers (100 μg/ml hygromycin B, 50 μg/ml chlorimuron ethyl, or 300 μg/ml phosphinothricin). No growth indicated successful excision of the LML cassettes. For further verification, the excision was examined with DNA sequencing of the related gene locus (see Supplemental Material Fig. S-3). If the fungus still retained the ability to grow on selection marker-containing medium, additional rounds of excision were conducted.

### RNA preparation and quantitative real-time PCR (qPCR)

About 20 mg of *T. reesei* mycelium was harvested. Total RNA was extracted using the FastRNA Pro Red Kit (MPbio, U. S. A.), according to the manufacturer’s instructions. Reverse transcription was performed with 1000 ng of total RNA using TransScript All-in-One First-Strand cDNA Synthesis SuperMix for qPCR (TransGen, China), according to the manufacturer’s instructions. For qPCR, the TransStart TipTop Green qPCR SuperMix (TransGen, China) was used with 200 nM of forward and reverse primers (see Supplemental Material Table S-4) and 1 μl of 10-fold diluted cDNA in a final volume of 20 μl. For *hpt* transcription analysis, a SYBR green assay with reference to a small GTPase gene (*sar*1) was performed. Thermocycling was performed in an ABI StepOne Plus thermocycler (Applied Biosystems, USA).

### Measurement of fluorescent protein

About 10^6^ conidia were inoculated into 10 mL MA containing 1% D-xylose, and cultured for 1 to 3 days. The mycelial suspension was collected and analyzed for the expression of fluorescent protein under a fluorescence microscope (Olympus BX50, Japan).

### Mutagen sensitivity

Sensitivity to UV exposure was determined as described^22^.

## Additional Information

**How to cite this article**: Zhang, L. *et al.* Light-inducible genetic engineering and control of non-homologous end-joining in industrial eukaryotic microorganisms: LML 3.0 and OFN 1.0. *Sci. Rep.* **6**, 20761; doi: 10.1038/srep20761 (2016).

## Supplementary Material

Supplementary Information

## Figures and Tables

**Table 1:** Self-excision colony counts of LML2.0a–e cassettes in three different filamentous fungi.

Cassette	Self-excision colonies (total colonies)^a^		
	*H*ypocrea *jecorina*	*Neurospora crassa*	*Aspergillus niger*
LML 2.0a	80(96)	84(96)	72(96)
LML 2.0b	78(96)	81(96)	72(96)
LML 2.0c	77(96)	75(96)	72(96)
LML 2.0d	76(96)	84(96)	21(96)
LML 2.0e	83(96)	37(96)	90(96)

^a^12 transformants, with 8 fungal isolates each.

**Table 2:** Total self-excision efficiencies of LML2.0a cassette with different carbon sources in *cre1-*deficient strain *Hypocrea jecorina* RUT C-30.

Carbon source	Self-excision colonies (total colonies)^a^	Percentage
D-xylose	80(96)	83.33%
Xylitol	10(96)	10.47%
L-arabinose	12(96)	12.50%
L-arabitol	9(96)	9.38%
Glycerol	27(96)	28.13%
Glucose	6(96)	6.25%

^a^12 transformants, with 8 fungal isolates each.

**Table 3:** Total self-excision efficiencies of LML2.1a–k cassettes in RUT C-30.

Cassette	Self-excision colonies (total colonies)^a^	Percentage
LML 2.1a	88(96)	91.67%
LML 2.1b	25(96)	26.04%
LML 2.1c	9(96)	9.38%
LML 2.1d	7(96)	7.29%
LML 2.1e	6(96)	6.25%
LML 2.1f	4(96)	4.17%
LML 2.1g	3(96)	3.13%
LML 2.1h	2(96)	2.08%
LML 2.1i	2(96)	2.08%
LML 2.1j	2(96)	2.08%
LML 2.1k	0(96)	0.00%

^a^12 transformants, with 8 fungal isolates each.

**Table 4:** Homologous integration and self-excision efficiencies of additional seven genes deleted in Qm6aΔ*tku70.*

Strain	Homologous integration colonies (total colonies)	Self-excision colonies (total colonies)		
		round 1	round 2	round 3
Qm6aΔ*tku70*Δ*cbh1*	8(8)	84(96)^a^	nd^d^	nd^d^
Qm6aΔ*tku70*Δ*cbh2*	8(8)	80(96)^a^	nd^d^	nd^d^
Qm6aΔ*tku70*Δ*xyn1*	8(8)	79(96)^a^	nd^d^	nd^d^
Qm6aΔ*tku70*Δ*cre1*	8(8)	85(96)^a^	nd^d^	nd^d^
Qm6aΔ*tku70*Δ*ace1*	7(8)	79(84)^b^	nd^d^	nd^d^
Qm6aΔ*tku70*Δ*ace2*	7(8)	48(84)^b^	nd^d^	nd^d^
Qm6aΔ*tku70*Δ*cbh1*Δ*cre1*	8(8)	89(96)^a^	nd^d^	nd^d^
Qm6aΔ*tku70*Δ2.1a-*xyr1*	4(4)	0(96)^c^	1(96)^c^	4(96)^c^
Qm6aΔ*tku70*Δ2.0a-*xyr1*	4(4)	0(96)^c^	2(96)^c^	6(96)^c^
Qm6aΔ*tku70*Δ2.11-*xyr1*	4(4)	6(96)^c^	19(96)^c^	35(96)^c^
Qm6aΔ*tku70*Δ2.12-*xyr1*	4(4)	6(96)^c^	22(96)^c^	32(96)^c^

^a^8 transformants, with 12 fungal isolates each;

^b^7 transformants, with 12 fungal isolates each;

^c^4 transformants, with 24 fungal isolates each;

^d^nd, no detection means not done.

**Table 5:** Self-excision colony counts of LML3.0 cassette.

Strain	Marker	Self-excision colonies(total colonies)^a^
*H*. *jecorina* Qm6a	hygromycin B	62(96)
Qm6aΔ*tku70*Δ*xyr1*	hygromycin B	64(96)
*Neurospora crassa*	hygromycin B	58(96)
*Aspergillus niger*	hygromycin B	44(96)
*Metarhizium anisopliae*	phosphinothricin	49(96)
*Metarhizium anisopliae*	chlorimuron ethyl	55(96)

^a^12 transformants, with 8 fungal isolates each.
