# Supplementary material for: Light-inducible genetic engineering and control of non-homologous end-joining in industrial eukaryotic microorganisms: LML 3.0 and OFN 1.0
Source: Sci Rep. 2016 Feb 9;6:20761. doi: 10.1038/srep20761 (PMC4746737; doi:10.1038/srep20761)
Supplement: Supplementary Information [file srep20761-s1.pdf]

Light-inducible genetic engineering and control of non-homologous end-joining in industrial eukaryotic microorganisms: LML 3.0 and OFN 1.0

Lei Zhang[1], Xihua Zhao[2], Guoxiu Zhang[1], Jiajia Zhang[1], Xuedong Wang[1], Suping Zhang[3], Wei Wang*[1], Dongzhi Wei*[1]

1. State key Lab of Bioreactor Engineering, New World Institute of Biotechnology, East China University of Science and Technology, Shanghai 200237, China.

2. College of Life Science, Jiangxi Normal University, Nanchang 330022, China

3. Research Center for Biomass Energy, East China University of Science and Technology, Shanghai 200237, China

***** Corresponding author: Wei Wang, Dongzhi Wei

Mailing address: East China University of Science and Technology, P.O.B. 311, 130 Meilong Road, Shanghai 200237, China

Phone: +86-21-64251923, Fax: +86-21-64250068.

E-mail: wadexp@ecust.edu.cn; dzhwei@ecust.edu.cn

Table S-1. Filamentous fungi strains constructed in this study.

Strain	Relevant features	Source
Hypocrea jecorina Qm6a	wide type strain	ATCC
H. jecorina RUT-C30	industrial strain	ATCC
Neurospora crassa FGSC2489	wild type strain	FGSC
Metarhizium anisopliae ARSEF2575	wild type strain	ARSEF
Aspergillus niger	wild type strain	ATCC
Qm6aΔ*tku70*	*H. jecorina* Qm6aΔ*tku70*	This study
Qm6aΔ*tku70*Δ*cbh1*	*H. jecorina* Qm6aΔ*tku70*Δ*cbh1*	This study
Qm6aΔ*tku70*Δ*cbh2*	*H. jecorina* Qm6aΔ*tku70*Δ*cbh2*	This study
Qm6aΔ*tku70*Δ*xyn1*	*H. jecorina* Qm6aΔ*tku70*Δ*xyn1*	This study
Qm6aΔ*tku70*Δ*cre1*	*H. jecorina* Qm6aΔ*tku70*Δ*cre1*	This study
Qm6aΔ*tku70*Δ*ace1*	*H. jecorina* Qm6aΔ*tku70*Δ*ace1*	This study

Qm6aΔ*tku70*Δ*ace2*	*H. jecorina* Qm6aΔ*tku70*Δ*ace2*	This study
Qm6aΔ*tku70*Δ*cbh1*Δ*cre1*	*H. jecorina* Qm6aΔ*tku70*Δ*cbh1*Δ*cre1*	This study
Qm6aΔ*tku70*Δ2.1a-*xyr1*	*H. jecorina* Qm6aΔ*tku70*Δ*xyr1*	This study
Qm6aΔ*tku70*Δ2.0a-*xyr1*	*H. jecorina* Qm6aΔ*tku70*Δ*xyr1*	This study
Qm6aΔ*tku70*Δ2.11-*xyr1*	*H. jecorina* Qm6aΔ*tku70*Δ*xyr1*	This study
Qm6aΔ*tku70*Δ2.12-*xyr1*	*H. jecorina* Qm6aΔ*tku70*Δ*xyr1*	This study
Qm6aΔ*tku70*&OFN 1.0A	*H. jecorina* Qm6Δ*tku70*a harboring OFN 1.0A cassette	This study
Qm6aΔ*tku70*&OFN 1.0B	*H. jecorina* Qm6aΔ*tku70* harboring OFN 1.0B cassette	This study
Qm6aΔ*tku70*&OFN 1.0C	*H. jecorina* Qm6aΔ*tku70* harboring OFN 1.0C cassette	This study
Qm6aΔ*tku70*&OFN 1.0D	*H. jecorina* Qm6aΔ*tku70* harboring OFN 1.0D cassette	This study
RUT C-30Δ*tku70*Δ*xyn1*::*z1cre-rfp*	*H. jecorina* RUT C-30Δ*tku70*Δ*xyn1*::*z1cre-rfp*	This study
RUT C-30Δ*tku70*Δ*xyn1*::*cre-rfp*	*H. jecorina* RUT C-30Δ*tku70*Δ*xyn1*::*cre-rfp*	This study

Table S-2. Plamids constructed in this study.

Plasmid	Relevant features	Source
LML 1.0	A copy of Steiger et al.'s study	Steiger et al., 2011
LML 2.0f	A failed 2.0 cassette	This study
LML 2.0a-k	Successful 2.0 cassettes	This study
LML 2.0s	A 2.0 cassette for imaging of marker self-excision	This study
LML 2.0a-bar	A 2.0a cassette harboring *bar* marker	This study
LML 2.0a-sur	A 2.0a cassette harboring *sur* marker	This study
LML 2.1a-i	2.1 cassettes containing both a LE mutant *lox* and a RE mutant *lox*	This study
LML 2.1j, k	2.1 cassettes containing double mutant *lox* sites	This study
LML 2.11	2.1a cassettes containing one *loxJT15-FRT* site and one *loxJTZ17-FRT* site	This study
LML 2.12	2.1a cassettes containing one *loxJT15-FRT* site and one *FRT-loxJTZ17* site	This study
LML 3.0	light-inducible self-excision cassette	This study

Del2.1a-*tku70*	*tku70* deletion using LML 2.1a cassette	This study
Del2.1a-*cbh1*	*cbh1* deletion using LML 2.1a cassette	This study
Del2.1a-*cbh2*	*cbh2* deletion using LML 2.1a cassette	This study
Del2.1a-*xyn1*	*xyn1* deletion using LML 2.1a cassette	This study
Del2.1a-*cre1*	*cre1* deletion using LML 2.1a cassette	This study
Del2.1a-*ace1*	*ace1* deletion using LML 2.1a cassette	This study
Del2.1a-*ace2*	*ace2* deletion using LML 2.1a cassette	This study
Del2.1a-*xyr1*	*xyr1* deletion using LML 2.1a cassette	This study
Del2.0a-*xyr1*	*xyr1* deletion using LML 2.0a cassette	This study
Del2.11-*xyr1*	*xyr1* deletion using LML 2.11 cassette	This study
Del2.12-*xyr1*	*xyr1* deletion using LML 2.12 cassette	This study
OFN 1.0A	An on-off control cassette of nonhomologous end-joining (NHEJ) pathway	This study
OFN 1.0B	An on-off control cassette of nonhomologous end-joining (NHEJ) pathway	This study

OFN 1.0C	An on-off control cassette of nonhomologous end-joining (NHEJ) pathway	This study
OFN 1.0D	An on-off control cassette of nonhomologous end-joining (NHEJ) pathway	This study
Δ*xyn1*::*z1cre-rfp*	*z1cre-rfp* expression cassette using *xyn1* promoter	This study
Δ*xyn1*::*cre-rfp*	*cre-rfp* expression cassette using *xyn1* promoter	This study

Table S-3. Primers used in this study.

Name	Sequences (5'-3')	Relevant gene
KU705-F	*GATTACGAATTCTTAATTAA*TCGCTCCATGACGGCTTGAAC	*tku70*
KU705-R	*TGCTATACGAATAATTTCTAGA*TCAGCACTTCGTCTTCGTCTTCG	
KU703-F	*ACTAGTGAGCTCATTT*CTTGGTGCGTGCTTCGATCTAAC	
KU703-R	*AGTGCCAAGCTTATTT*CCTCTGTGAATACTCGGCAACCA	
CBH15-F	*GATTACGAATTCTTAATTAA*CCATCATCACGCACGACCACTT	*cbh1*
CBH15-R	*TGCTATACGAATAATTTCTAGA*TGCTACTAGACACTGCTATCGGTG	
CBH13-F	*ACTAGTGAGCTCATTT*ATCTCCGCGAATCTCCTCTTCTC	

CBH13-R	*AGTGCCAAGCTTATTT*GCTACCATGACTGTCACGATAGAGA	
CBH25-F	*GATTACGAATTCTTAATTAA*AGATGCTGGAGTCTGGTGTAAT	
CBH25-R	*TGCTATACGAATAATTTCTAGA*GAGATATAAGGCAGAATGGATACGA	*cbh2*
CBH23-F	*ACTAGTGAGCTCATTT*GTAGATTCCAATTACTCCACCTCTT	
CBH23-R	*AGTGCCAAGCTTATTT*CAATGGTTCACCGCCTTATGT	
tHe5-F	*GATTACGAATTCTTAATTAA*CTGAGATGACGGGTTCTGATGTTGT	
tHe5-R	*TGCTATACGAATAATTTCTAGA*CACGAGCTGGTTGGCAATGGAT	*tre108087*
tHe3-F	*ACTAGTGAGCTCATTT*AACGCACAGGTTCGCAGACG	
tHe3-R	*AGTGCCAAGCTTATTT*CGACGCATACTCACGGCTTCAT	
CRE15-F	*GATTACGAATTCTTAATTAA*GAAATGGAAGCCATTGGAGCACG	
CRE15-R	*TGCTATACGAATAATTTCTAGA*GAGCGGCAGTCAAAAAGCAAGTAC	*cre1*
CRE13-F	*ACTAGTGAGCTCATTT*CTGCCAATGTAGGTAAGTAGTAAGG	
CRE13-R	*AGTGCCAAGCTTATTT*GACGAGGACAAGGAAGGAGTT	

ACE15-F	*GATTACGAATTCTTAATTAA*TTTGCGGTGACTTCATCGTACAAG	*ace1*
ACE15-R	*TGCTATACGAATAATTTCTAGA*GACCGCATGGCGGCCGAGATCTGT	
ACE13-F	*ACTAGTGAGCTCATTT*AAAGATTGCGACACATACAATGA	
ACE13-R	*AGTGCCAAGCTTATTT*CATTAGACCGACCTAATTACTTACT	
ACE25-F	*GATTACGAATTCTTAATTAA*TAGTTTGGACTAAAGGCCTCCCT	*ace2*
ACE25-R	*TGCTATACGAATAATTTCTAGA*TCAGAAGGGAATCGCGATGAT	
ACE23-F	*ACTAGTGAGCTCATTT*GGTTGACCAGGTGAAGGATGAT	
ACE23-R	*AGTGCCAAGCTTATTT*ACTAGTACTCGGTACGTGATGCT	
XYR15-F	<u>TTAATTAAGTTAACTCTAGA</u>ACGAGTATCTCCGAAATTCCCTTTGG	*xyr1*
XYR15-R	<u>ACTAGT</u>GCGCTGTGTGCGATGTGAAG	
XYR13-F	<u>TCTAGA</u>GGAGGCCACTCAATCGTATGACG	
XYR13-R	<u>ATTTAAATGAGCTCACTAGT</u>GAACCTCTTACTCACATTCACTTGACTTG	

Table S-4. qPCR primers used in this study.

Name	Sequences (5’-3’)	Relevant gene
KU70-F	GGCTGACGACCTGCGAGATAT	*tku70*
KU70-R	GGAGGATCTTGTAGTGCCATTGC	
KU70-arm-F	CGAGTCGCATCTGGTCTGACA	
KU70-arm-R	ACGGACTGACTTCAATGGCTAGAA	
CBH1-arm-F	TCGCAGTAGCAGGCACTCATT	*cbh1*
CBH1-arm-R	GGTACGGAACAGTTATGTCCAAGC	
CBH2-arm-F	TCTCCTCGCCTAATACCTGTGAAG	*cbh2*

CBH2-arm-R	CTGGTCGTGTTACTCCTCCTGTTA	
tHe-arm-F	AGCATCACCACCACCACCAA	*tre108087*
tHe-arm-R	AGAGCGACGCACGGCAATT	
CRE1-arm-F	CACTCACTCACACACCCTCACA	*cre1*
CRE1-arm-R	CTAGTATGCGGTGGTCCTCGTT	
ACE1-arm-F	TCCTTGCCATCTCCTCCTCCT	*ace1*
ACE1-arm-R	CGAACAGAAGCGACCACCAATT	
ACE2-arm-F	GCGATGGAGGCGATCACGATA	*ace2*
ACE2-arm-R	ATGGACGGCGATGTTCTTTGC	
XYR1-arm-F	GTCCAATCCTCTCCGTCGCTAT	*xyr1*
XYR1-arm-R	GGCTGTTGCCGAATGTGTTGA	

DNA sequences of LML 2.1a

CTATGACATGATTACGAATTCTTAATTAAGTTAACTCTAGAAATTATTCGTATAGCATACA
TTATACGAAGTTATACTAGAgatatcAAGCAACTACGTAAAACTCCATGAGATTGCAGATG
CGGCCCACTGGAATACAACATCCTCCGCAAGTCCGACATGAAGCCCCTTGACTTGATT
GGCAGGCTAAATGCGACATCTTAGCCGGATGCACCCCAGATCTGGGAACGCGCCGCT
TGAGGCCCGAAGCGCCGGGTTCGATGCATTACTGCCATATTTCAGCAGTTAACTAGGAC
CGGCTTGTGTCGATATTGCGGGTGGCGTTCAATCTATTCCGGCACTCCTATGCCGTTTGA
TCCGATACCTGGAGGGCGTGCTTTAGGCAAAATGCCAAGCTTCGAGGATACTGTACGA
GCCGCTTTCAACCTCACTTGATGATGTCTGAGTTTCATCAAGAGAATTGAAGTCAAAG
CTCAAATCATGATGTGAAGAGGTTTTGAATGTGGAAGAATTCTGCATATATAAAGCCAT
GGAAGAAGACGTAAAACTGAGACAGCAAGCTCAACTGCATAGTATCGACTTCAAGGA
AAACACGCACAAATAATCATCATGGCCcacgtgATGACCCGACGTCGATGCAACTCATTGA
GAGTCCTTATGTATGCTTACATCCAGAATGGTCGTCAATCCAGCCGAGCGATTCACGGA
ACTTGTACGCCGGATGAGGCTGGTCCCGTCTGCCACTTTCACCTGAGCGTCTTCAACTC
GGCGACCTACAGCTCGGCAGTCAAGAGTAGGTAAGGACGGCATAGTACCACTACTAGG
TACTAGCATTCTCTTCGGGCAATATTTACTTACTACCTACCTAGACTAGGGTACCCTATC
GAGCTCGCCCCCGTCAAGCAGACCCTGAACTTCGACCTGCTGAAGCTGGCCGGCGAC
GTCGAGAGCAACCCCGGCCCCgatAACGACAAGAAGCGCAAGAGCTGGGGCCAGGTC
CTGCCCGAGCCCAAGACCAACCTGCCCCCCCGCAAGCGCGCCAAGACCTCCAATTTAC
TGACCGTACACCAAAATTTGCCTGCATTACCGGTCGATGCAACGAGTGATGAGGTTCG
CAAGAACCTGATGGACATGTTCAGGGATCGCCAGGCGTTTTCTGAGCATACCTGGAAA
ATGCTTCTGTCCGTTTGCCGGTCGTGGGCGGCATGGTGCAAGTTGAATAACCGGAAAT
GGTTTCCCGCAGAACCTGAAGATGTTCGCGATTATCTTCTATATCTTCAGGCGCGCGGT
CTGGCAGTAAAAACTATCCAGCAACATTTGGGCCAGCTAAACATGCTTCATCGTCGGTC
CGGGCTGCCACGACCAAGTGACAGCAATGCTGTTTCACTGGTTATGCGGCGGATCCGA
AAAGAAAACGTTGATGCCGGTGAACGTGCAAAACAGGCTCTAGCGTTCGAACGCACT
GATTTCGACCAGGTTCGTTCACTCATGGAAAATAGCGATCGCTGCCAGGATATACGTAA
TCTGGCATTTCTGGGGATTGCTTATAACACCCTGTTACGTATAGCCGAAATTGCCAGGAT
CAGGGTTAAAGATATCTCACGTACTGACGGTGGGAGAATGTTAATCCATATTGGCAGAA
CGAAAACGCTGGTTAGCACCGCAGGTGTAGAGAAGGCACTTAGCCTGGGGGTAACTA
AACTGGTCGAGCGATGGATTTCCGTCTCTGGTGTAGCTGATGATCCGAATAACTACCTG
TTTTGCCGGGTCAGAAAAAATGGTGTTGCCGCGCCATCTGCCACCAGCCAGCTATCAA
CTCGCGCCCTGGAAGGGATTTTTGAAGCAACTCATCGATTGATTTACGGCGCTAAGGAT
GACTCTGGTCAGAGATACCTGGCCTGGTCTGGACACAGTGCCCGTGTCGGAGCCGCGC
GAGATATGGCCCGCGCTGGAGTTTCAATACCGGAGATCATGCAAGCTGGTGGCTGGAC
CAATGTAAATATTGTCATGAACTATATCCGTAACCTGGATAGTGAAACAGGGGCAATGG
TGCGCCTGCTGGAAGATGGCGATTAGcgcgcgcgCcaccaccaccaccaccactaatagGATCGTTCAA
ACATTTGGCAATAAAGTTTCTTAAGATTGAATCCTGTTGCCGGTCTTGCGATGATTATCA
TATAATTTCTGTTGAATTACGTTAAGCATGTAATAATTAACATGTAATGCATGACGTTATT
TATGAGATGGGTTTTTATGATTAGAGTCCCGCAATTATACATTTAATACGCGATAGAAAA
CAAAATATAGCGCGCAAACTAGGATAAATTATCGCGCGCCGTGTCATCTATGTTACTAGA
TCACTAGAGAGCTCTGTACAGTGACCGGTGACTCTTTCTGGCATGCGGAGAGACGGAC
GGACGCAGAGAGAAGGGCTGAGTAATAAGCGCCACTGCGCCAGACAGCTCTGGCGGC

TCTGAGGTGCAGTGGATGATTATTAATCCGGGACCGGCCGCCCCTCCGCCCCGAAGTG
GAAAGGCTGGTGTGCCCCTCGTTGACCAAGAATCTATTGCATCATCGGAGAATATGGA
GCTTCATCGAATCACCGGCAGTAAGCGAAGGAGAATGTGAAGCCAGGGGTGTATAGCC
GTCGGCGAAATAGCATGCCATTAACCTAGGTACAGAAGTCCAATTGCTTCCGATCTGGT
AAAAGATTCACGAGATAGTACCTTCTCCGAAGTAGGTAGAGCGAGTACCCGGCGCGTA
AGCTCCCTAATTGGCCCATCCGGCATCTGTAGGGCGTCCAAATATCGTGCCTCTCCTGC
TTTGCCCGGTGTATGAAACCGGAAAGGCCGCTCAGGAGCTGGCCAGCGGCGCAGACC
GGGAACACAAGCTGGCAGTCGACCCATCCGGTGCTCTGCACTCGACCTGCTGAGGTC
CCTCAGTCCCTGGTAGGCAGCTTTGCCCCGTCTGTCCGCCCGGTGTGTCGGCGGGGTT
GACAAGGTCGTTGCGTCAGTCCAACATTTGTTGCCATATTTTCCTGCTCTCCCCACCAG
CTGCTCTTTTCTTTTCTCTTTCTTTTCCCATCTTCAGTATATTCATCTTCCCATCCAAGAA
CCTTTATTTCCCCTAAGTAAGTACTTTGCTACATCCATACTCCATCCTTCCCATCCCTTAT
TCCTTTGAACCTTTCAGTTCGAGCTTTCCCACTTCATCGCAGCTTGACTAACAGCTACC
CCGCTTGAGCAGACATCACCATGCCTGAACTCACCGCGACGTCTGTCGAGAAGTTTCT
GATCGAAAAGTTCGACAGCGTCTCCGACCTGATGCAGCTCTCGGAGGGCGAAGAATCT
CGTGCTTTCAGCTTCGATGTAGGAGGGCGTGGATATGTCCTGCGGGTAAATAGCTGCGC
CGATGGTTTCTACAAAGATCGTTATGTTTATCGGCACTTTGCATCGGCCGCGCTCCCGAT
TCCGGAAGTGCTTGACATTGGGGAATTCAGCGAGAGCCTGACCTATTGCATCTCCCGC
CGTGCACAGGGTGTCACGTTGCAAGACCTGCCTGAAACCGAACTGCCCGCTGTTCTGC
AGCCGGTCGCGGAGGCCATGGATGCGATCGCTGCGGCCGATCTTAGCCAGACGAGCGG
GTTCGGCCCATTCGGACCGCAAGGAATCGGTCAATACACTACATGGCGTGATTTCATAT
GCGCGATTGCTGATCCCCATGTGTATCACTGGCAAACTGTGATGGACGACACCGTCAGT
GCGTCCGTCGCGCAGGCTCTCGATGAGCTGATGCTTTGGGCCGAGGACTGCCCCGAAG
TCCGGCACCTCGTGCACGCGGATTTCGGCTCCAACAATGTCCTGACGGACAATGGCCG
CATAACAGCGGTCATTGACTGGAGCGAGGCGATGTTCGGGGATTCCCAATACGAGGTC
GCCAACATCTTCTTCTGGAGGCCGTGGTTGGCTTGTATGGAGCAGCAGACGCGCTACT
TCGAGCGGAGGCATCCGGAGCTTGCAGGATCGCCGCGGCTCCGGGCGTATATGCTCCG
CATTGGTCTTGACCAACTCTATCAGAGCTTGGTTGACGGCAATTTCGATGATGCAGCTT
GGGCGCAGGGTCGATGCGACGCAATCGTCCGATCCGGAGCCGGGACTTTCGGGCGTAC
ACAAATCGCCCGCAGAAGCGCGGCCGTCTGGACCGATGGCTGTGTAGAAGTACTCGCC
GATAGTGGAAACCGACGCCCCAGCACTCGTCCGAGGGCAAAGGAATAGAGTAGATGC
CGACCGGGATCCACTTAACGTTACTGAAATCATCAAACAGCTTGACGAATCTGGATATA
AGATCGTTGGTGTCGATGTCAGCTCCGGAGTTGAGACAAATGGTGTTCAGGATCTCGAT
AAGATACGTTCATTTGTCCAAGCAGCAAAGAGTGCCTTCTAGTGATTTAATAGCTCCAT
GTCAACAAGAATAAAACGCGTTTCGGGTTTACCTCTTCCAGATACAGCTCATCTGCAAT
GCATTAATGCATTGGACCTCGCAACCCTAGTACGCCCTTCAGGCTCCGGCGAAGCAGA
AGAATAGCTTAGCAGAGTCTATTTTCATTTTCGGGAGACGAGATCAAGCAGATCAACG
GTCGTCAAGAGACCTACGAGACTGAGGAATCCGCTCTTGGCTCCACGCGACTATATATT
TGTCTCTAATTGTACTTTGACATGCTCCTCTTCTTTACTCTGATAGCTTGACTATGAAAAT
TCCGTCACCAGCCCCTGGGTTCGCAAAGATAATTGCACTGTTTCTTCCTTGAACTCTCA
AGCCTACAGGACACACATTCATCGTAGGTATAAACCTCGAAAATCATTCCTACTAAGAT
GGGTATACAATAGTAACCATGCATGGTTGCCTAGTGAATGCTCCGTAACACCCAATACG
CCGGCCGAAACTTTTTTACAACTCTCCTATGAGTCGTTTACCCAGAATGCACAGGTACA
CTTGTTTAGAGGTAATCCTTCTTACTAGAATAACTTCGTATAGCATACATTATAGCAATTT

ATACTAGTGAGCTCATTTAAATAAGCTTGGCACTGGCCGTCGTTTT

Fig. S-1

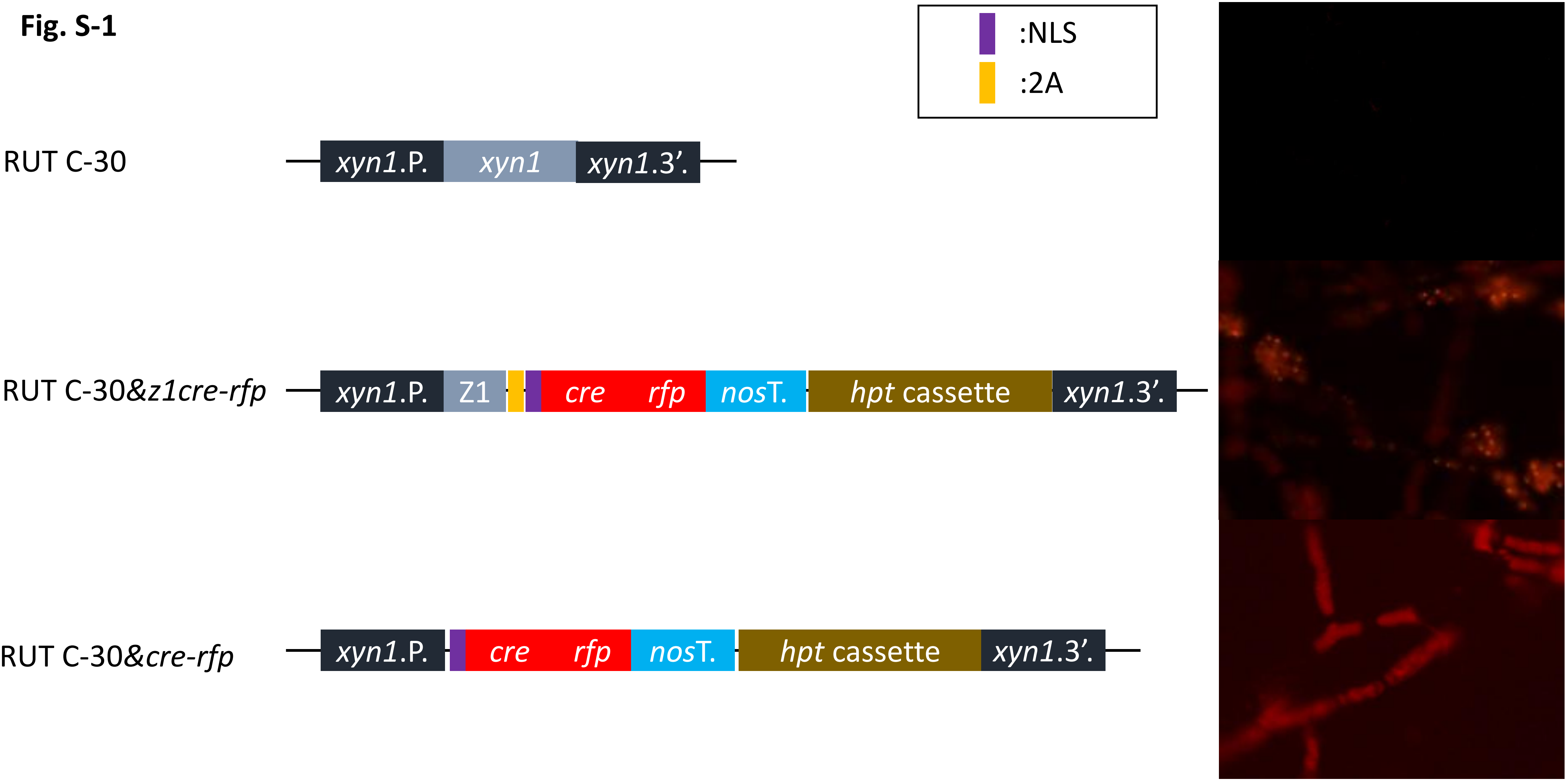

Fig. S-1. Expression cassettes of two chimeric genes, *z1cre-rfp* and *cre-rf*, their corresponding transformants RUT C-30&*z1cre-rfp* and RUT C-30&*cre-rfp* analyzed for the expression of fluorescent protein under a fluorescence microscope. The higher fluorescence intensity is observed in the nucleus as bright red dots in RUT C-30&*z1cre-rfp* than that of RUT C-30&*cre-rfp.*

Fig. S-2

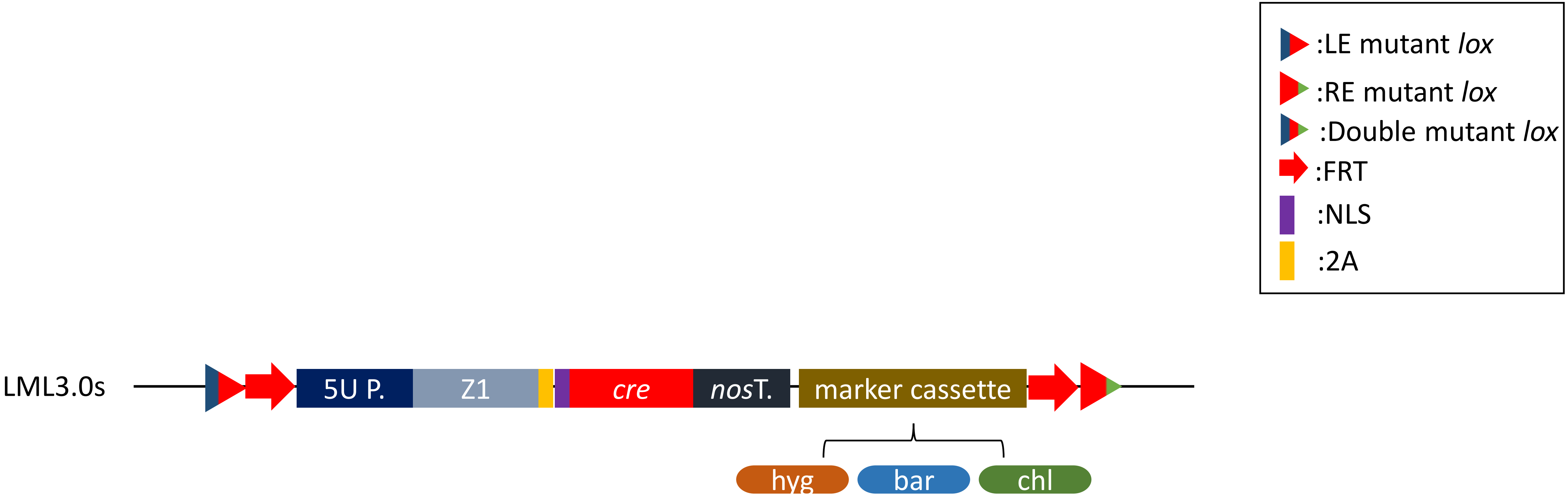
:LE mutant lox
:RE mutant lox
:Double mutant lox
:FRT
:NLS
:2A
LML3.0s
5U P.
Z1
cre
nosT.
marker cassette
hyg
bar
chl

Fig. S-3

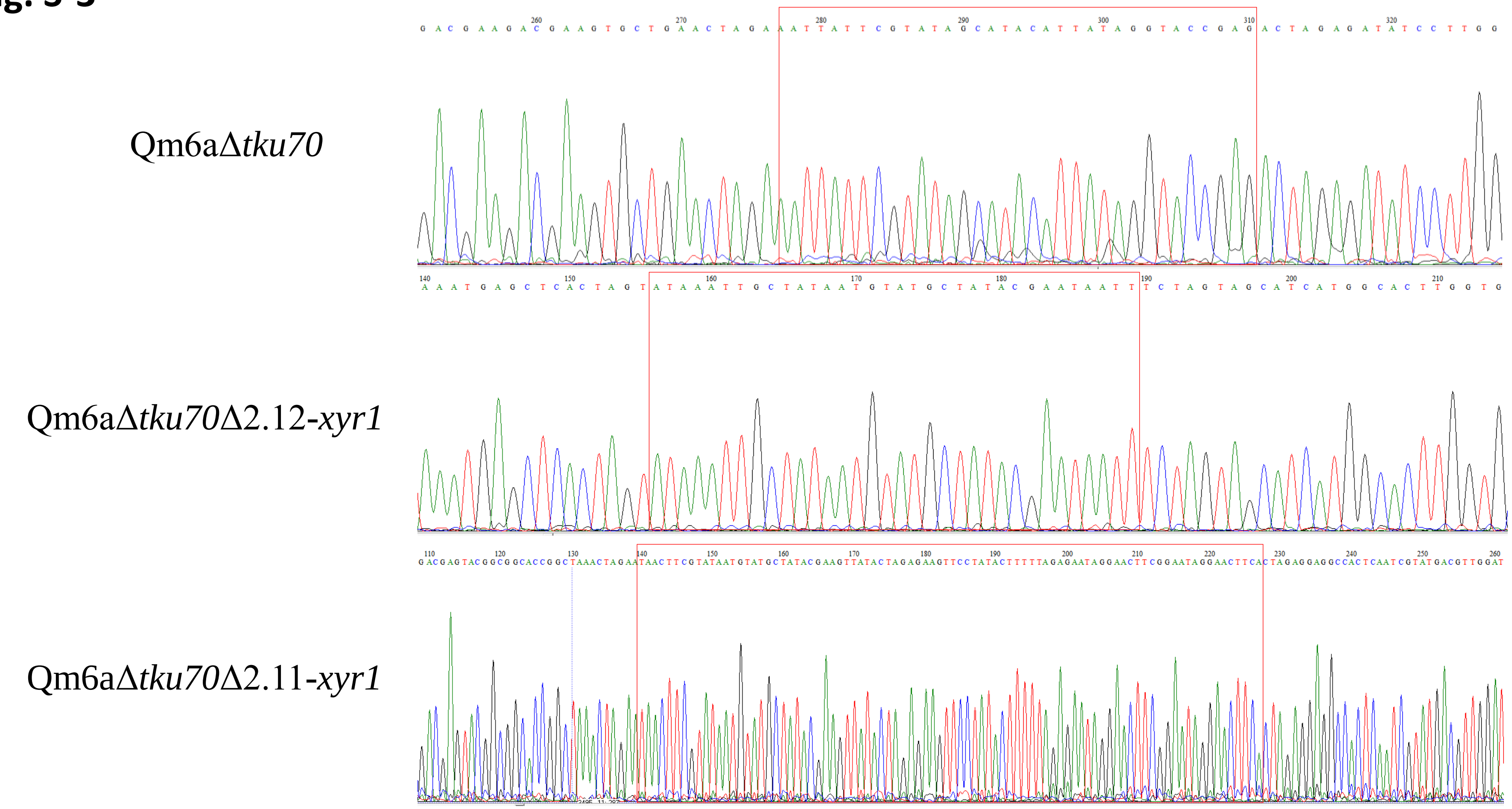

Fig. S-3. Sequencing analysis of self-excision at the related gene locus. The left *lox* or *lox-FRT* sites after self-excision are showed in red sqare.
